# Readiness Assessment of Healthcare Professionals to Integrate Mental Health Services into Primary Healthcare of Persons with Skin-Neglected Tropical Diseases in Ghana: A Structural Equation Modeling

**DOI:** 10.3390/ijerph22070991

**Published:** 2025-06-23

**Authors:** Samuel Adjorlolo, Stephanopoulos Kofi Junior Osei, Emma Efua Adimado, Mawuko Setordzi, Vincent Valentine Akorli, Lawrencia Obenewaa Aprekua, Paul Kwame Adjorlolo

**Affiliations:** 1 Department of Mental Health, School of Nursing and Midwifery, University of Ghana, Legon, Accra P.O. Box LG 43, Ghana; 2 Research and Grant Institute of Ghana, Legon, Accra P.O. Box LG 1004, Ghana; mwksetordzi3@gmail.com; 3 Department of Epidemiology, School of Public Health, University of Ghana, Accra P.O. Box LG 13, Ghana; stephanpaosei@gmail.com; 4 Department of Research, Education and Administration, School of Nursing and Midwifery, University of Ghana, Legon, Accra P.O. Box LG 43, Ghana; edmypumpkin85@gmail.com; 5 School of Nursing and Midwifery, University of Health and Allied Sciences, Ho-Volta Region, Ho PMB 31, Ghana; valentineakorli@gmail.com; 6 Department of Psychology, School of Social Sciences, University of Ghana, Accra P.O. Box LG 44, Ghana; lawsenyah@gmail.com; 7 Department of Biostatistics, School of Public Health, University of Ghana, Accra P.O. Box LG 13, Ghana; padjorlolo@gmail.com

**Keywords:** skin-NTD, mental health, integrated care, healthcare professionals, Ghana

## Abstract

The burden of mental health among individuals affected by skin-neglected tropical diseases (skin-NTDs) has increased significantly, prompting systemic measures to improve their mental health and well-being. Healthcare professionals have instrumental roles to play in this area in terms of integrating mental health into the existing primary and community healthcare services for skin-NTDs. The current study investigates the readiness of healthcare professionals for integrated healthcare, barriers to mental health service delivery and the professional development needs for mental health service delivery. A total of 252 healthcare professionals recruited from Nkwanta North and South Districts in the Oti Region of Ghana participated in the study by completing a set of questionnaires measuring the above variables, in addition to demographic factors. Descriptive statistics were used to summarize the study variables while Pearson Moment Product Correlation was used to investigate the relationship between continuous study variables. Confirmatory factor analysis (CFA) was conducted to elucidate the factorial validity of the study measures. Structural Equation Modeling (SEM) was used to examine the association between the variables and the mediating effects of professional development needs. The results showed that over 50% of the participants encountered several barriers in their attempt to provide mental health services to patients, and over 80% of them requested training and capacity building in mental health. CFA supports a two-factor structure of the readiness scale and one-factor structure of mental health barrier and professional development needs scales. SEM revealed a significant relationship between readiness for integrated healthcare, mental health barriers and professional development needs (*p* < 0.05). Further SEM analysis revealed that professional development needs significantly mediated the relationship between readiness for integrated healthcare and mental health barriers (*p* < 0.05). Addressing mental health professional development needs of healthcare professionals will help ensure their readiness for integrated healthcare for people with skin-NTDs.

## 1. Introduction

Neglected tropical diseases (NTDs) are a group of heterogeneous diseases commonly reported in areas with poor access to clean water and safe ways to dispose of human waste [1]. Some NTDs such as buruli ulcer, leprosy and lymphatic filariasis are collectively called skin-NTDs because they manifest on the skin or visible areas of the body. For the past two decades, skin-NTDs have been recognized as major drivers of psychosocial morbidity and mental burden among affected individuals and their caregivers [2]. Human resource, budgetary and organizational challenges have negatively affected efforts to address mental health issues in this vulnerable population, particularly in Ghana and other low- and middle-income countries. The integration of mental health services into primary health has been proposed as a promising strategy to address mental health treatment [3]. Empowering health professionals to provide basic mental health services such as screening and counseling to individuals seeking healthcare services is a crucial component of integrated healthcare models. However, for integrated health, the readiness of healthcare professionals and their institutions, operationalized as ability to deliver services at a specified minimum standard, is extremely important [4]. The current study investigates the readiness of healthcare professionals to integrate mental health into primary healthcare for people with skin-NTD in Ghana.

### 1.1. The Burden of Mental Health in Skin-NTDs

Individuals affected by skin-NTDs suffer multiple agonies in terms of (1) their “ugly and scary” appearance which makes them distinct from other community members; (2) the attribution of the causes of the diseases to witchcraft, ancestral curse and/or punishment from God/gods which fuels rejection, discrimination and exclusion from community practices and social services [5,6]. These negative behaviors potentiate the bidirectional experience and development of mental health problems and stigma [5]. However, until recent as 2012, there was a near-neglect of mental health burden in skin-NTD [2]. The existing literature has pointed to an increasing prevalence of mental health issues among individuals affected by skin-NTDs, globally. For example, the prevalence of depression in lymphatic filariasis was estimated to be as high as 97% in South India, Asia [7] and 70% in Togo, West Africa [8]. Similar findings have been reported among individuals affected by leprosy [9] and cutaneous leishmaniasis [10]. Among those with active and past buruli ulcer infection in Ghana, depression and anxiety issues were estimated at 27% and 42%, respectively [11]. In a more recent systematic review, Adjorlolo and colleagues reported a high mental health burden, with the prevalence of depression, stress and anxiety estimated to range from 7 to 54%, 8–43%, and 19–53%, respectively [6]. The burden of mental health have been implicated in poor involvement in health, social and community services and activities, diminished education and vocational opportunities, and restrictions in the exercise of civil rights [9]. These observations provide impetus to address the burden of mental health in skin-NTD.

### 1.2. Addressing the Burden of Mental Health in Skin-NTD: Integrated Healthcare

Interventions to improve the mental health of individuals affected by skin-NTDs are limited in Ghana due to a myriad of challenges such as the uneven distribution of psychiatric hospitals, inadequate human resources, leading to a widening treatment gap, financial constraints and inadequate hospital beds, supplies and psychotropic medicines [12,13,14,15,16]. Ongoing efforts to decentralize mental health services across the various levels of healthcare delivery have similarly been fraught with human resources, logistics and financial challenges. The proposal for integrated healthcare was intended to reduce the above challenges by ensuring access to mental health services at the basic level of healthcare. Inherent in the proposal is empowering non-mental health professionals, through training and resource provision, to deliver basic mental health and psychosocial support to patients. This is intended to reorient the healthcare system to integrate mental health services into the primary and community healthcare services often delivered to patients with physical health conditions [17]. This initiative is intended to promote and improve access to mental health services, anchored in several core international agreements and frameworks, such as the WHO Roadmap on Neglected Tropical Diseases 2021–2030, WHO Mental Health Action Plan 2013–2030, WHO Mental Health Gap Action Programme and WHO Quality Rights initiative [18]. Mental Health Policy 2019–2030 of the Ministry of Health, Ghana, emphasized integrated healthcare whereby mental health services are incorporated seamlessly into the general healthcare system using a human rights approach. This evidence-based model will enable sustainable healthcare system strengthening by leveraging existing resources [17]. Ultimately, this will contribute to reducing the morbidity, disability and psychosocial effects of skin-NTD through a person-centered approach. As mentioned previously, the readiness of healthcare professionals for integrated healthcare is crucial.

### 1.3. Readiness of Healthcare Professionals for Integrated Healthcare

Healthcare professionals such as nurses and community disease control officers are instrumental in addressing mental health issues among individuals affected by skin-NTDs. These professionals are highly skilled in establishing therapeutic relationships with patients; a relationship that can be utilized to promote mental health through the screening and administration of low-cost interventions [2]. Anecdotally, individuals affected by skin-NTDs hardly seek mental health services on their own, suggesting that the involvement of healthcare professionals in providing mental health services is crucial. For example, these professionals could be entrusted with the responsibility of screening and assessing the mental states of this vulnerable population. The benefits associated with integrating mental health programs into existing primary and community healthcare services have been well documented [3,19,20], strongly suggesting that individuals living with skin-NTDs will equally benefit from any integrated program. However, the fundamental and unaddressed question in the skin-NTD literature and more importantly in the Ghanaian context relates to the readiness of healthcare professionals to integrate mental health services into primary and community healthcare systems.

As stated previously, readiness broadly refers to the psychological and behavioral preparedness of organizational members to implement organization change [21]. Readiness for integrated healthcare has been identified as an important issue [22], with evidence suggesting that healthcare professionals are likely to show interest and participate in integrated healthcare when there is a high sense of readiness at the organizational and individual levels [23]. In contrast, poor organizational readiness often leads to resistance to any planned change, as such changes are interpreted as undesirable and burdensome [24]. Consequently, understanding readiness for integrated healthcare from the perspectives of healthcare professionals is crucial to inform interventions to empower these professionals. While this can be understood from multiple sources, a focus on how readiness is associated with the barriers to providing mental health services and the mental health professional development needs of healthcare professionals will support healthcare administrators and policy makers to improve readiness for integrated care. These practice and development issues are essential ingredients for readiness examination given that they can support capacity building initiatives, and resource allocation/prioritization. To this end, the current study is aimed at elucidating the readiness of healthcare professionals to integrate mental health into primary healthcare system, taking into consideration barriers affecting the provision of mental health services by healthcare professionals, their professional development needs for integrated healthcare, and the demographic factors influencing their readiness.

### 1.4. Study Objectives

1. Examine the influence of demographic characteristics, previous training on mental health and provision of mental health services on readiness to integrate mental health into primary care for people with skin-NTD.
2. Elucidate the relationship between mental health barriers, professional development needs and readiness to integrate mental health into primary care for people with skin-NTD.
3. Investigate the mediating role of professional development needs on the relationship between mental health barriers and readiness to integrate mental health into primary care for persons with skin-NTD.

## 2. Method

### 2.1. Study Design and Setting

A questionnaire was used to collect data from 252 healthcare professionals using a cross-sectional survey design. The study was conducted at the Nkwanta North and South Districts in the Oti Region of Ghana as part of a larger study designed to integrate mental health into primary and community care of individuals affected by skin-NTDs. These two districts were purposely selected because they are known to be co-endemic for skin-NTDs like Buruli ulcer and onchocerciasis. Healthcare professionals in these districts can help to improve the mental health and well-being of individuals affected by skin-NTDs.

### 2.2. Study Participants and Sample Size

The study population comprised healthcare professionals, such as nurses, midwives, physician assistants and medical officers working in the Nkwanta South and North Districts in the Oti region. Participants must have worked in the above Districts for at least 6 months prior to the commencement of the study to ensure that they have some experiences regarding the provision of healthcare services to persons with skin-NTDs. The convenience sampling method was used to select participants based on their availability and willingness to participate in the study. They were recruited from various levels of healthcare, including district hospitals, health centers and community health planning service (CHPS) compounds, to ensure a diverse representation of the target population. A power analysis, using a G*power computer program [25], indicates a minimum sample of 234 participants to detect medium effects (f^2^ = 0.26) with 80% power and 0.05 alpha. The total sample size recruited was 252 participants.

### 2.3. Data Collection Measures

*Organizational Readiness for Implementing Change* (ORIC) [24] was used to measure organizational readiness to integrate mental health into primary and community care for persons living with skin-NTDs and related disease conditions. The ORIC was developed based on Weiner’s theory of organization, and it consists of 10 items that were generated from a pool of items following the application of rigorous statistical measures [24]. The items are rated on a four-point Likert scale, ranging from strongly disagree [1] to strongly agree [5]. The ORIC total score is obtained by summing the individual items, with higher scores reflecting readiness for change. The ORIC consists of two subscales, namely the Change Commitment Scale and the Change Efficacy Scale. Each subscale has five (5) items. The ORIC was subjected to CFA to confirm its factor structure in Ghanaian samples. The ORIC tool demonstrated good reliability and validity indices in a previous study [24,26]. The inter-class correlation (ICC) of its sub-scales’ ranged from 0.51 to 0.98 [24] Another study reported high internal consistency for the change commitment (Cronbach’s α = 0.91) and change efficacy sub-scales (Cronbach’s α = 0.94) subscales. The tool has EFA factor loadings ranging from 0.55 to 0.92 [24]. The CFA findings in the current study are summarized in Table 1.

*The Mental Health Barriers Scale (MHBS)* was obtained from a previous questionnaire used to ascertain the barriers to providing maternal mental health services in Ghana [27]. The questionnaire was developed following an extensive review of the literature and consultations with healthcare professionals and academics. A total of 6 items were culled to index the MHBS. The items are scored on a 5-point Likert-type scale, ranging from 1 (i.e., strongly disagree)–5 (strongly agree). The total score on the MHBS ranges from 6 to 30, with high scores indicating high perceived barriers. The MHBS has also demonstrated high reliability (Cronbach’s α = 0.88) in a Ghanaian context [27]. The tool further exhibited satisfactory factor loadings, ranging from 0.31 to 0.62. The CFA findings in the current study are summarized in Table 1.

*The Professional Development Needs Scale* (PDNS) was developed by extracting salient items from prior questionnaires used to assess maternal mental health learning needs among nurses and midwives in Ghana [28]. A total of 8 items were deemed relevant for the study following extensive deliberation among the study team members and consultation with healthcare practitioners in the study setting. The 8-items are rated on a 5-point Likert scale, ranging from 1 (i.e., strongly disagree) to 5 (strongly agree). A total score was calculated by summing the participants’ responses on the various items, with higher scores indicating more learning needs. In a previous Ghanaian study, the PDNS demonstrated a Cronbach’s alpha of 0.80 [28] The CFA findings in the current study are summarized in Table 1.

*Demographic and professional background measures:* The study also collected demographic and background characteristic data including age, gender, years of professional practice, level of education, participation in mental-health related continuous professional development programs, facility type of participants and experience with mental health assessments.

### 2.4. Procedure for Data Collection

The questionnaires were administered to a cross-section of healthcare professionals based on their availability and willingness to participate in the study. Field officers were recruited from Nkwanta South and North Districts and trained to assist in the administration of the questionnaires. Regular supervision and monitoring of the data collection process was conducted to address any pressing issues. At each healthcare facility, the research team approached the healthcare professionals on duty (i.e., morning or afternoon) for their consent. Prior to that, the participants were briefed on the study goals and objectives and their responsibilities as research participants. Research ethics principles, including those relating to confidentiality and anonymity were observed. Out of the 260 questionnaires distributed, 252 were returned, representing a response rate of 96%. The data collection spanned June to July 2023. The study received ethics approval from the Ghana Health Service Ethics Review Committee (GHS-ERC: 023/03/23).

### 2.5. Data Analysis

Data were analyzed using IBM SPSS Statistics for Windows (version 27) (SPSS Inc, Chicago, IL, USA) and IBM SPSS AMOS 27 (IBM Corp., Armonk, NY, USA). The demographic characteristics of the participants were summarized using descriptive statistics. Mean-level analyses involving independent sample *t*-tests and analysis of variance (ANOVA) were conducted to examine demographic differences in readiness to integrate mental health into primary healthcare. Significant ANOVA results were followed up with Bonferroni-adjusted post hoc analysis. A series of confirmatory factor analyses (CFA) was conducted to investigate the factor structure of the study measures using the maximum likelihood estimation method in Analysis of Moment Structures (AMOS) software, version 26. Structural Equation Modeling (SEM) was implemented to investigate the relationship between readiness for integrated mental healthcare, mental health barriers and professional development needs. The SEM tested for the mediation effect of professional development needs using a bootstrapping technique, a nonparametric procedure for resampling that does not require the assumption of a normal sample distribution [29]. SEM allows for the identification of both direct and indirect effects while providing data on the overall fit of the model. Indirect effects were evaluated at the 95% bias-corrected percentile, following 5000 bootstrapping iterations.

The fits of the models were evaluated using the chi-square test. However, because the chi-square value is influenced by sample size, its ratio (CMIN) to the degree of freedom (df) was used, with a CMIN/df value of <3, indicating an acceptable fit between the hypothesized model and sample data [30]. The following fit indicators [31] were also used to evaluate the various models: the comparative fit index (CFI; ≥0.90 = adequate; ≥0.95 = good), Tucker–Lewis index (TLI; ≥0.90 = adequate, ≥0.95 = good), goodness of fit index (GFI; ≥0.90 adequate) and root mean square error of approximation with its 90% confidence interval (RMSEA; 0.10≤ = acceptable. ≤0.08 = adequate, and ≤0.05 = good).

## 3. Results

### 3.1. Demographic Characteristics of Participants

A total of 252 participants took part in the study (Table 2), comprising registered general nurses (48.4%), enrolled nurses (30.2%) and others. The average age of the participants was 30 (SD = 4.85). They had practiced for an average of 4 years (SD = 2.86). More than half were males (52.8%) and graduates from diploma-awarding institutions (52%). Close to half of the participants (46.8%) had not participated in continuous professional development training in mental health. About 80% had enquired about the mental health of patients with skin-NTDs and other medical conditions.

*Mental Health Barriers and Professional Development Needs of Healthcare Professionals.*

The barriers to mental health practice and the mental health professional development needs of healthcare professionals are summarized in Table 3. As can be seen, the weights associated with the barrier items differ; however, the overall findings point to more barriers, with more than half of the participants endorsing each of the barriers. Similar findings were observed for professional development needs whereby about 80% of the participants agreed/strongly agreed that they need training and capacity building in salient areas relating to mental health and mental healthcare.

### 3.2. Demographic Influence on Readiness to Integrate Mental Health into Primary Healthcare

Readiness to integrate mental health into primary healthcare did not differ across gender and educational level (*p* ≥ 0.05). In contrast, individuals who participated in at least one continuous professional development program in mental health scored significantly higher than those who did not on commitment (*M* = 20.86, *SD* = 3.19 vs. M = 19.79, *SD* = 2.63), efficacy (*M* = 20.66, *SD* = 2.99 vs. M = 19.11, *SD* = 3.02) and overall readiness (*M* = 41.52, *SD* = 5.70 vs. M = 38.90, *SD* = 4.49) to integrate mental health into primary healthcare (*p* < 0.01). ANOVA indicated a significant effect of enquiring about the mental health of patients on commitment, [*f*(3, 248) = 5.75, *p* < 0.001], efficacy [*f*(3, 248) = 3.12, *p* = 0.027] and overall readiness [*f*(3, 248) = 4.66, *p* = 0.003]. Bonferroni-adjusted post hoc analysis revealed that participants who enquired about the mental health of patients ≥ 5 times were more likely to be ready for integrated mental healthcare (*p* < 0.05).

### 3.3. Correlations Between Study Variables

Table 4 contains a summary of the intercorrelations between study variables. The Organizational Readiness for Integrated Healthcare Scale and its subscales correlated significantly (*p* < 0.01). They also correlated with the professional development needs of healthcare professionals to provide mental health services (*p* < 0.01). However, only change commitment correlated significantly and negatively with barriers to providing mental health services (MHBS; *p* < 0.05).

### 3.4. Confirmatory Factors Analysis (CFA) of Readiness, Barriers and Professional Development Needs

**Figure 1:**
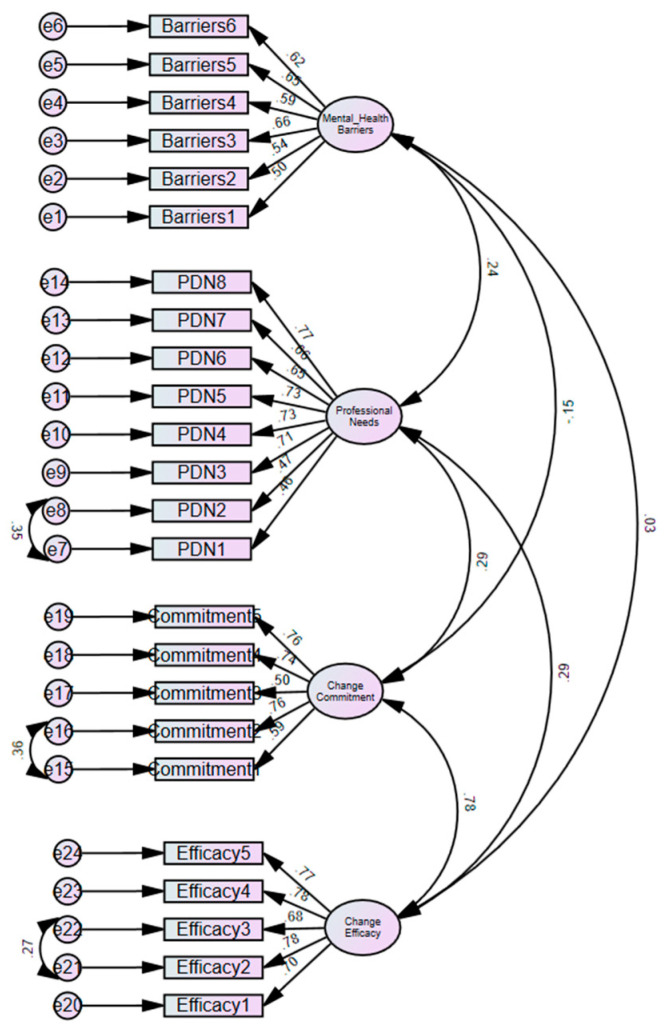
Confirmatory factor analysis of the study variables.

The result of CFA of the measurement model, comprising readiness (i.e., commitment and efficacy), barriers and professional development needs, is summarized in Figure 1. Following a series of re-specifications, whereby error variances of some manifest variables were allowed to correlate on their respect latent variables, the final model provided an adequate fit to the data: (χ^2^ (243) = 405.04, *p* < 0.001; CMIN/df = 1.67; TLI = 0.92; CFI = 0.93; RMSEA = 0.05). The hypothesized paths were significant (*p* < 0.05), except for barriers, commitment and efficacy. The factor loadings of each manifest variable were significant (*p* < 0.01) and ranged from 0.46 to 0.78.

### 3.5. Relationship Between Readiness, Barriers and Professional Development Needs

**Figure 2:**
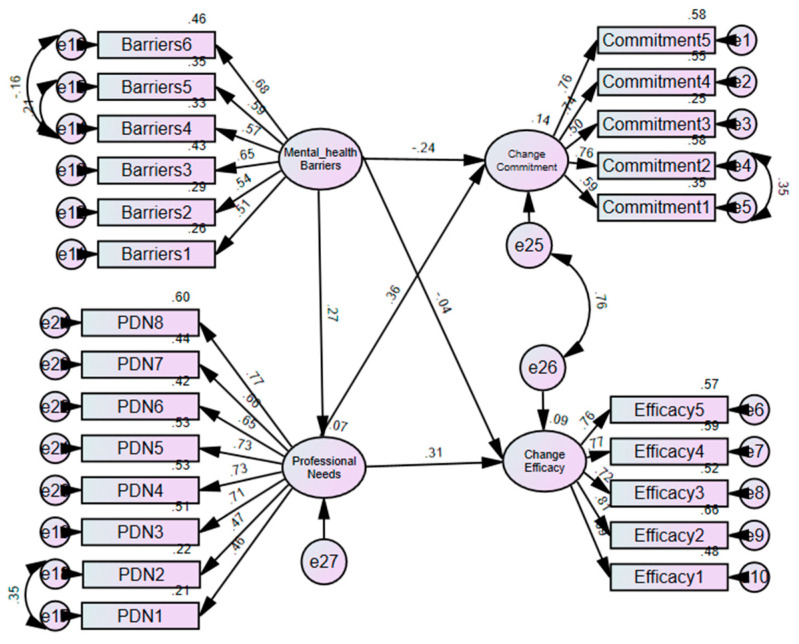
Structural Equation Modeling and mediation analysis of the relationship between professional needs, mental health barriers and readiness for change.

SEM was conducted to assess the relationships among the study variables: readiness, barriers and professional development needs. Figure 2 is a summary of the final model, showing the various factors and their interrelations, as well as factor loadings. The final structural model demonstrated an acceptable fit to the data following the re-specification of the initial model by allowing error variances of some items to correlate freely: (χ^2^ (242) = 402.60, *p* < 0.001; CMIN/df = 1.66; TLI = 0.92; CFI = 0.93; RMSEA = 0.05). The hypothesized paths were significant (*p* < 0.05), with the exception of barriers and commitment to integrated care (*p* = 0.583). The factor loadings were all significant (*p* < 0.001), ranging from 0.51 to 0.68 for barriers; 0.46 to 0.77 for professional development needs; 0.69 to 0.82 for efficacy and 0.59 to 0.74 for commitment.

A summary of the SEM mediation analysis is provided in Table 5. As can be seen, the indirect effect of professional development needs on the relationship between perceived barriers to providing mental health services and commitment to integrate mental health into primary care was significant and positive (*b* = 0.078, *p* = 0.011). Similarly, the direct effect of perceived barriers on commitment to integrate mental health into existing primary care was significant (*b* = −0.192, *t* = −2.77, *p* = 0.006), suggesting that professional development needs partially mediated the relationship.

The relationship between perceived barriers to providing mental health services and efficacy to integrate mental health into primary care was indirectly influenced by professional development needs of healthcare providers (*b* = 0.084, *p* = 0.009). Further analyses revealed that the direct effect of perceived barriers on efficacy for integrated care was statistically significant (*b* = −0.043, *t* = −0.549, *p* = 0.583). It follows that professional development needs fully mediated the relationship between the perceived barriers and efficacy of integrated care.

## 4. Discussion

Integrated healthcare delivery is a promising approach to universal and decentralized healthcare that aligns with the leave-no-one-behind agenda by the United Nation’s Sustainable Development Goals (Agenda 2030). The burden of mental health among individuals affected by skin-NTD has led to calls for mental health to be integrated into the primary and community healthcare services available to this vulnerable population. This is largely because skin-NTDs are associated with significant mental health and psychological distress [6]. While several indicators have been proposed to understand the integration of behavioral health into primary healthcare, assessing the readiness of healthcare professionals for integrated healthcare is a quintessential requirement, particularly as it relates to challenges affecting the provision of mental health services and the professional development needs of healthcare professionals to provide mental health services. The current study provided initial evidence on this topical issue.

There were several barriers affecting the ability of healthcare professionals to provide mental health services to patients, including those affected by skin-NTDs. These barriers are similar to those reported by previous studies involving non-skin-NTD populations, suggesting a somewhat general problem across the healthcare system [27,32]. A critical examination of the barriers largely suggests that they emanate from (1) individual sources (e.g., lack of skills to initiate conversation about mental health), (2) organizational domain (e.g., heavy workload at the facility) and system-level sources (e.g., lack of resources to screen for mental health issues). This observation is largely consistent with previous findings on barriers affecting the provision of mental health services by non-mental health professionals [33,34]. Clearly, the study findings suggest that the concept of task-shifting/sharing proposed by the WHO and partners to address human resources issues and increase access to mental health services is seriously threatened. It is therefore unsurprising that the participants registered their professional development needs, cutting across salient areas of mental health knowledge and skills. In an earlier studies, participants expressed enthusiasm for training and capacity building programs to reposition them to provide mental health and psychological services [27]. The importance of training and capacity building in mental health is strengthened by the study finding.

Readiness for integrated healthcare is the beginning stage of implementing a change necessary to extend mental health services to vulnerable populations, including individuals affected by skin-NTDs. Assessing the barriers to mental health service delivery and the capacity development needs of healthcare professionals to overcome the barriers is extremely important due to their interconnectedness with the readiness to integrate mental health into the primary and community care of individuals affected by skin-NTDs. The direction of the correlations between these factors, as with previous studies [35,36,37], is overly instructive, indicating that the presence of barriers will negate efforts to integrate mental health services into primary healthcare while training and capacity development initiatives have the tendency to increase the readiness of these professionals. The mediating influence of mental health capacity development needs between barriers to mental health services and readiness for integrated healthcare further highlights the importance of the call for deliberate and targeted intervention programming to enhance the knowledge, skills and competencies of healthcare professionals to administer mental health interventions. This is further strengthened by the study finding that suggests that healthcare professionals who participated in at least one training program were more ready to integrate mental health into primary healthcare. The demographic characteristics of the participants such as education, professional category (e.g., nurse versus physician assistant), gender and category of healthcare facility (e.g., District hospital versus CHPS compound) did not influence readiness for integrated healthcare, in contrast with previous findings [28]. Our finding is not surprising given that the training programs for the various cadres of non-mental health professionals in Ghana pay less attention to mental health [27,28]. While some training programs dedicate a semester to training students in mental health, others combine mental health training with behavioral and social sciences such as sociology, thereby restricting capacity building efforts in mental health. The major learning is that the demographic characteristics of the study participants may not significantly influence the choice and type of interventions to promote their readiness for integrated healthcare.

From the foregoing, there is a pressing need to intervene to ensure that mental health services are available to individuals affected by skin-NTD. In Ghana, this can be accomplished through short-term capacity building programs in mental health or through a restructuring of the curriculum of healthcare institutions to incorporate salient topics in mental health. A deliberate national policy and directives to the various health training institutions to include integrated healthcare training into their curriculum would certainly change the knowledgebase and competence of healthcare professionals to extend mental health services to people affected by skin-NTDs. Organizational and system-level resources are equally needed to support readiness for integrated healthcare. These include the provision of tools to screen and assess mental health problems and guidelines on referral systems and follow-ups. The existing national referral guidelines should be revised to accommodate mental health services, as the current focus on emergency and related services restricts its application in various contexts. Overall, focusing on the professional development needs of healthcare professionals will facilitate readiness for integrated healthcare that incorporates both physical and mental health services for people living with skin-NTDs.

### Limitations of the Study

The study findings should be evaluated in light of the following limitations. The study participants were conveniently sampled based on their availability at the time of the study, creating the possibility that others with different experiences may have been excluded from the study. In effect, this may restrict the extension of the findings beyond the study participants/settings. Second, the study variables were measured with self-report questionnaires. Although the use of questionnaires is a common practice in knowledge generation, there is no mechanism to check the accuracy of participant responses or detect any bias in the responses obtained from the participants. Notwithstanding the above, our study findings on barriers to mental health services and capacity development needs of healthcare providers do not differ systematically from those reported in the literature. Nevertheless, future studies should aim to address the limitations stated above.

## 5. Conclusions

The quest to provide integrated healthcare is underpinned by several international and national frameworks and policy guidelines, including Ghana’s Mental Health Policy 2019–2030. At the core of the call for integration is the readiness of healthcare professionals and their institutions to incorporate mental health services into the existing healthcare services at the community, primary, secondary, etc., levels. The study findings provide timely information to support efforts and discussions to operationalize integrated healthcare, particularly for people affected by skin-NTDs. It is imperative to understand the existing practice system as it relates to the provision of mental health services. This includes understanding the factors that impede the provision of mental health services across multiple levels, such as provider factors (e.g., knowledge), institutional (e.g., availability of screening resources) and national-level factors (e.g., referral systems). Understanding the barriers should be followed by measures to address them, which necessitate exploring the professional development needs of healthcare professionals with respect to integrated healthcare. In conclusion, the integration of mental health services into the primary healthcare system can be accomplished granted that healthcare professionals demonstrate readiness for the same.

## Figures and Tables

**Table 1:** Summary of factor loadings and CFA Results of the data collection measures.

S. No	Scales and Their Items	Factor Loadings
	**Organizational Readiness to Implement Change** (χ^2^ (32) = 69.92, *p* < 0.001; CMIN/df = 2.19; TLI = 0.95; CFI = 0.97; RMSEA = 0.06)	
	Change Commitment Scale	
1	We are committed to implementing this change	0.57
2	We are determined to implement this change.	0.76
3	We are motivated to implement this change.	0.49
4	We will do whatever it takes to implement this change.	0.74
5	We want to implement this change.	0.77
	Change Efficacy Scale	
1	We feel confident that they can manage the politics of implementing this change.	0.69
2	We feel confident that the organization can support people as they adjust to this change.	0.78
3	We feel confident that they can coordinate tasks so that implementation goes smoothly.	0.68
4	We feel confident that they can keep track of progress in implementing this change	0.78
5	We feel confident that they can handle the challenges that might arise in implementing this change.	0.77
	**Mental Health Barriers Scale** (χ^2^ (8) = 14.82, *p* = 0.063; CMIN/df = 1.85; TLI = 0.96; CFI = 0.98; RMSEA = 0.05)	
	*….*	
1	Heavy workload at the unit/department.	0.50
2	Lack of knowledge about mental health issues.	0.55
3	Lack of measures to detect mental health issues.	0.67
4	Time allocated to see patients is too short.	0.54
5	Lack the skills to start conversation about mental health.	0.62
6	Lack of mental health resources to serve as a guide or reference.	0.63
	**Professional Development Needs Scale** (χ^2^ (19) = 25.11, *p* = 0.157; CMIN/df = 1.32; TLI = 0.99; CFI = 0.99; RMSEA = 0.03).	
	I need education on	
1	The relationship between NTD and mental health	0.46
2	Commonly diagnosed mental health conditions in NTDs	0.47
3	Screening and assessment of mental health issues	0.71
4	Relationship building with persons with mental health problems.	0.73
5	Working with families of people with mental health problems.	0.73
6	Provision of low-cost psychosocial interventions	0.65
7	Therapeutic communication	0.66
8	Referring and following up on patients with mental health problems	0.78

**Table 2:** Summary of participants demographic characteristics.

Variables	Frequency	Percentage
Gender		
Male	133	52.8
Female	119	47.2
Marital status		
Single	142	56.3
Married	108	42.9
Education attainment		
Certificate	88	34.9
Diploma	131	52
Degree	33	13.1
Participated in Mental health CPD		
Not at all	118	46.8
Only once	59	23.4
2 to 4 times	49	19.4
≥5 times	26	10.3
Professional background		
Registered general nurse	122	48.4
Enrolled nurse	76	30.2
Physician assistant	8	3.2
Medical officer	3	1.2
Midwife	15	6
Community health nurse	13	5.2
Other	15	6
Type of health facility		
Hospital	70	27.8
Health center	83	32.9
CHPS compound	89	35.3
Other	10	4
Enquired about mental health		
Not at all	44	17.5
Only once	84	33.3
2 to 4 times	73	29
≥5 times	51	20.2

**Table 3:** Summary of barriers and professional development needs of healthcare professionals to integrate mental health into primary healthcare.

S. No		Strongly Disagree n (%)	Disagree n (%)	Neutral n (%)	Agree n (%)	Strongly Agree n (%)
	**Mental Health Barriers Scale**					
1	Heavy workload at the unit/department.	18 (7.1)	70 (27.8)	21(8.3)	112 (44.4)	31(12.3)
2	Lack of knowledge about mental health issues.	22 (8.7)	72 (28.6)	24 (9.5)	106 (42.1)	28 (11.1)
3	Lack of measures to detect mental health issues.	19 (7.5)	43 (17.1)	33 (13.1)	129 (51.2)	28 (11.1)
4	Time allocated to see patients is too short.	40 (15.9)	78 (31.0)	24 (9.5)	90 (35.7)	20 (7.9)
5	Lack the skills to start conversation about mental health.	29 (11.5)	74 (29.4)	20 (7.9)	111 (44.0)	18 (7.1)
6	Lack of mental health resources to serve as a guide or reference.	12 (4.8)	28 (11.1)	12 (4.8)	116 (46.0)	84 (33.3)
	**Professional Development Needs Scale**					
1	The relationship between NTD and mental health	5 (2.0)	10 (4.0)	16 (6.3)	158 (62.7)	63 (25)
2	Commonly diagnosed mental health conditions in NTDs	4 (1.6)	15 (6.0)	18 (7.1)	162 (64.3)	53 (21.0)
3	Screening and assessment of mental health issues	2 (0.8)	14 (5.6)	9 (3.6)	159 (63.1)	68 (27.0)
4	Relationship building with persons with mental health problems.	6 (2.4)	11 (4.4)	15 (6.0)	164 (65.1)	56 (22.2)
5	Working with families of people with mental health problems.	6 (2.4)	23 (9.1)	17 (6.7)	138 (54.8)	68 (27.0)
6	Provision of low-cost psychosocial interventions	3 (1.2)	11 (4.4)	20 (7.9)	147 (58.3)	71 (28.2)
7	Therapeutic communication	5 (2.0)	16 (6.3)	15 (6.0)	141 (56.0)	75 (29.8)
8	Referring and following up on patients with mental health problems	6 (2.4)	14 (5.6)	10 (4.0)	132 (52.4)	90 (35.7)

**Table 4:** Summary of intercorrelations, descriptive statistics and internal consistency of study measures.

	Commitment	Efficacy	Total Readiness	MHBS	PDNS
**Readiness**					
Commitment	1				
Efficacy	0.63 **	1			
Total Readiness	0.90 **	0.91 **	1		
**MHBS**	−0.14 *	0.01	−0.07	1	
**PDNS**	0.27 **	0.24 **	0.28 **	0.18 **	1
**Years of practice**	0.17 **	0.14 *	0.17 **	−0.06	0.19 **
Mean	20.36	19.94	40.29	19.73	32.34
SD	2.98	3.10	5.49	4.85	4.80
α	0.80	0.86	0.88	0.76	0.86

** = *p* < 0.01, * = *p* < 0.05. MHBS = Mental Health Barrier Scale; PDNS = Professional Development Needs Scale; Organizational Readiness for Implementing Change (ORIC).

**Table 5:** Test for mediation using a bootstrap analysis with a 95% confidence interval.

Relationships	Direct Effect	Indirect Effect	Confidence	Interval	*p*-Value	Conclusion
			Lower	Upper		
Barriers → Needs → Change commitment	−0.192 (−2.77)	0.078	0.019	0.192	0.011	Partial mediation
Barriers → Needs → Change efficacy	−0.043 (−0.549)	0.084	0.017	0.208	0.009	Full mediation

Note: Unstandardized coefficients reported. Values in parentheses are t-values. Bootstrap sample = 5000 with replacement. Barriers = perceived barriers; needs = professional development needs.

## Data Availability

Data generated or analyzed during this study can be found at https://doi.org/10.17632/ww22mnk9nk.1.
